# Treatment with MG132 prevents spontaneous activation of rat oocyte in culture and promotes embryonic development after intracytoplasmic sperm injection

**DOI:** 10.1038/s41598-022-06714-6

**Published:** 2022-02-17

**Authors:** Yuki Nakagawa, Takehito Kaneko

**Affiliations:** 1 Department of Chemistry and Biological Sciences, Faculty of Science and Engineering, Iwate University, Morioka, Iwate 020-8551 Japan; 2 Division of Fundamental and Applied Sciences, Graduate School of Science and Engineering, Iwate University, Morioka, Iwate 020-8551 Japan

**Keywords:** Biotechnology, Animal biotechnology

## Abstract

Intracytoplasmic sperm injection (ICSI) is an effective reproductive technique for obtaining rat offspring using preserved sperm with low or no motility. However, rat oocytes undergo spontaneous activation immediately after retrieval from the oviduct and poorly develop after ICSI unless it is performed quickly. Here, we evaluated whether treatment with MG132, the proteasome inhibitor, suppresses the spontaneous activation of oocytes before and during ICSI. After retrieval from the oviducts, the rate of development into morula and blastocyst from the oocytes cultured in vitro for 1 h prior to ICSI significantly decreased compared with that from the control oocytes subject to ICSI without culture (7% versus 36%). However, a higher proportion of oocytes treated with MG132 for 0, 1, and 3 h before and during ICSI developed into morulae and blastocysts (70%, 60%, and 52%, respectively). Offspring were obtained from oocytes treated with MG132 for 0 and 1 h before and during ICSI (percentage: 31%). Altogether, MG132 could suppress the spontaneous activation of rat oocytes and increase embryonic development after ICSI.

## Introduction

Rats have been widely employed as model organisms to gain a better understanding of human diseases^1,2^. Owing to advances in the production of genetically modified rats using genome editing tools, such as the clustered regularly interspaced short palindromic repeat (CRISPR) and CRISPR-associated protein 9 (Cas9) system^3–7^, the number of rat strains has been rapidly increasing in recent years. Sperm preservation is a simple method for maintaining various rat strains as genetic resources at a low cost. Offspring can be obtained from preserved sperm that has sufficient motility to penetrate oocytes using artificial insemination (AI) or in vitro fertilization (IVF). However, rat sperm are extremely sensitive to various physical stresses compared to other mammalian sperm^8^. Therefore, it is often impossible to fertilize oocytes with preserved sperm using AI or IVF as there is a decline in sperm motility during sperm handling. Intracytoplasmic sperm injection (ICSI) is an essential reproductive technique for the fertilization of oocytes with preserved sperm with low or no motility. In rats, normal offspring can be obtained from oocytes injected with both frozen^9–13^ and freeze-dried sperm^12–15^. To date, however, only few research groups have reported the successful production of rat offspring using ICSI due to the technical difficulty of rat ICSI.

Spontaneous activation of oocytes is one of the main causes of the difficulty in producing rat offspring through ICSI. In mammals, ovulated oocytes are temporarily arrested at metaphase II (MII) in the oviducts until fertilization^16^. However, rat oocytes collected from the oviduct spontaneously activate, and then progress to the metaphase III-like stage after extrusion of the second polar body without pronuclear formation^17–20^. Spontaneous activation occurs immediately in vitro, and more than half of the rat oocytes spontaneously activate within 70 min after separation from animal^21^. The spontaneous activation of rat oocytes before fertilization adversely affects subsequent embryonic development^22^. In fact, the fertilization and cleavage rates of rat embryos were found to significantly decrease by in vitro culture for 3 or 5 h before ICSI^22^. For successful rat ICSI, sperm must be quickly injected into oocytes before the spontaneous activation of oocytes. In previous reports, rat offspring could be stably obtained by completion of ICSI within 1 h after removal of cumulus cells^9–11,14^. However, the present protocol of rat ICSI requires a long period for the injection of sperm into oocytes as sperm heads must be individually picked up and injected into oocytes^9^. Suppressing the spontaneous activation of oocytes before and during ICSI may thus improve the efficiency of rat ICSI.

Several approaches have been used to suppress the spontaneous activation of rat oocytes^19,20^. Treatment with MG132, a proteasome inhibitor, is one of the strategies employed to suppress spontaneous activation. MG132 maintains a high activity of maturation promoting factor (MPF) and stops cell division by preventing the proteasomal degradation of cyclin B^23^. Previous reports revealed that MG132 could improve the efficiency of somatic cell nuclear transfer in rats by suppressing the spontaneous activation of recipient oocytes^24–26^. However, to our knowledge, no studies have reported the suppressive effect of MG132 on the spontaneous activation of oocytes before and during ICSI, and the embryonic development after ICSI.

Here, we evaluated the suppressive effect of the proteasome inhibitor, MG132, on the spontaneous activation of oocytes before and during ICSI.

## Results

### Embryonic development from oocytes collected at different times after human chronic gonadotropin (hCG) injection

First, we determined the effect of the time from the hCG injection to ICSI on the embryonic development from oocytes. Oocytes were collected from the oviducts of mature females at 18, 22, or 26 h after hCG injection. These oocytes were injected with freeze-thawed sperm within 1 h of retrieval, and fertilized oocytes were cultured in vitro to the blastocyst stage.

As shown in Table 1, 75–83% of oocytes survived after ICSI in all experiments, and more than 92% of the surviving oocytes were fertilized. There was no significant difference in the rate of development into morula and blastocyst between oocytes collected at 18 and 22 h after hCG injection (38% and 40%, respectively). However, this rate was significantly decreased in oocytes collected at 26 h after hCG injection (11%). Therefore, we used the oocytes collected at 18 h after hCG injection in the following experiments.

**Table 1:** Post-ICSI development of oocytes collected at different times after hCG injection.

Time (h) after hCG injection	No. (%) of oocytes			No. (%) of oocytes developed to		
	Injected	Survived^a^	Fertilized^b^	2-cell^c^	4-cell^c^	Morula/Blastocyst^cd^
18	87	65 (75)	61 (94)	56 (92)	43 (70)	23 (38)^e^
22	76	59 (78)	55 (93)	46 (84)	34 (62)	22 (40)^e^
26	95	79 (83)	73 (92)	56 (77)	15 (21)	8 (11)^f^

^a^Percentages based on the no. of oocytes injected.

^b^Percentages based on the no. of oocytes survived.

^c^Percentages based on the no. of oocytes fertilized. Embryonic development was estimated at day 1 (2-cell), day3 (4-cell) and day5 (Morula/Blastocyst) after ICSI (day 0).

^d^Percentages were significantly different at *P* < 0.05 (e vs. f).

### Evaluation of the spontaneous activation of oocytes during culture in vitro

**Figure 1:**
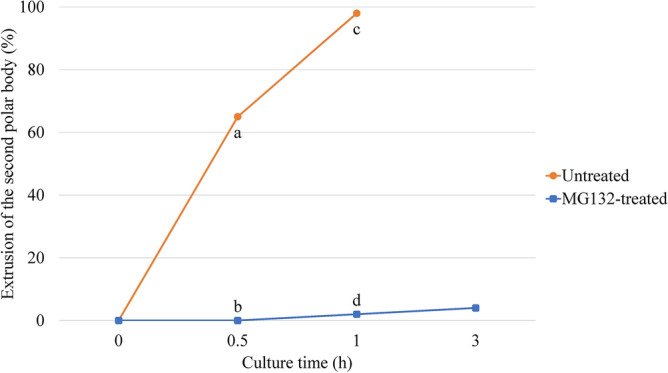
Suppressive effect of MG132 on the spontaneous activation of oocytes. Untreated: oocytes were cultured without MG132. MG132-treated: oocytes were cultured with MG132. Significant difference at *P* < 0.05 (a vs. b, c vs. d).

The effect of MG132 on the spontaneous activation of rat oocytes was determined. Briefly, oocytes were collected at 18 h after hCG injection, based on the results shown in Table 1. Oocytes at the MII stage were cultured in vitro in modified Krebs Ringer Bicarbonate (mKRB)^27^ with or without 5 μM MG132 for 3 h, and oocytes with the second polar body were considered to be activated (Fig. 1).

In the untreated group (n = 51), 98% of the oocytes extruded the second polar body within 1 h of culture. However, in the MG132-treated group (n = 52), only 4% of oocytes had the second polar body at 3 h of culture. At each time point of culture, the extrusion rate of the second polar body in MG132-treated oocytes was significantly lower than that in untreated oocytes.

### Embryo development from oocytes treated with MG132 before and during ICSI.

**Figure 2:**
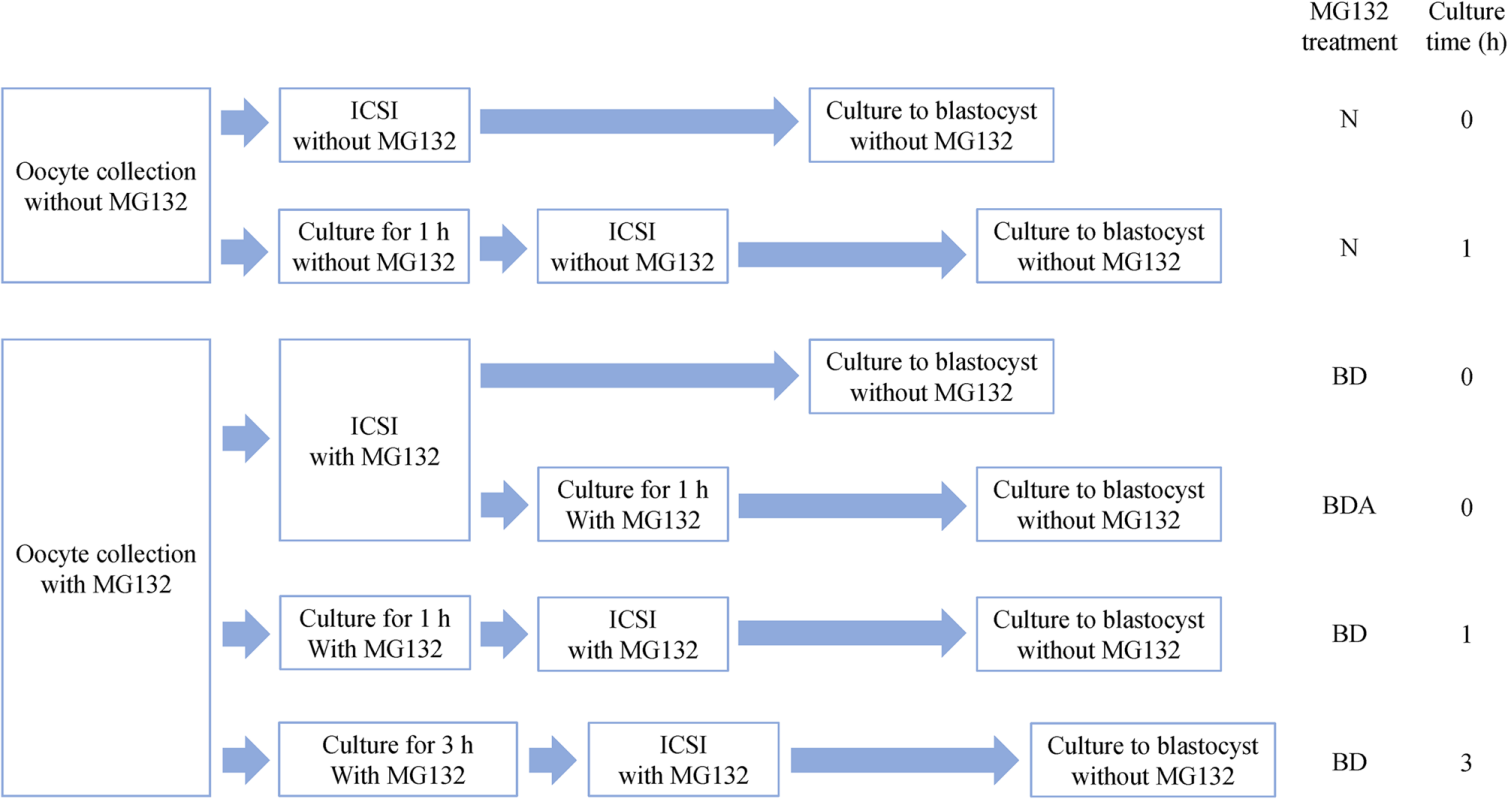
Time schedules for rat ICSI with MG132. N: Oocytes were untreated. BD: Oocytes were treated with MG132 before and during ICSI. BDA: Oocytes were treated with MG132 before, during, and 1 h after ICSI. Oocytes were cultured for 0, 1, or 3 h before ICSI.

The suppressive effect of MG132 on the spontaneous activation of oocytes before and during ICSI is presented in Table 2. We performed ICSI with MG132 according to the time schedule shown in Fig. 2. Oocytes were collected in mKRB with 5 μM MG132 at 18 h after hCG injection. The oocytes were cultured in mKRB containing 5 μM MG132 at 37.5 °C under 5% CO_2_ in air. The oocytes cultured for 0, 1, or 3 h were injected with freeze-thawed sperm in the presence of 5 μM MG132. The oocytes cultured for 0, 1, or 3 h were then injected with freeze-thawed sperm in the presence of 5 μM MG132. These oocytes were then transferred into fresh mKRB without MG132 immediately after ICSI. Some oocytes after ICSI with MG132 were further cultured in mKRB with 5 μM MG132 for 1 h, and transferred into mKRB without MG132. As a control, untreated oocytes were injected with sperm after culture in mKRB without MG132 for 0 or 1 h; culture in vitro was performed until the blastocyst stage. Almost all untreated oocytes cultured in mKRB without MG132 for 1 h underwent spontaneous activation before ICSI.

**Table 2:** Suppressive effect of MG132 on the spontaneous activation of oocytes and the in vitro development of oocytes injected with sperm.

MG132 treatment^a^	Culture time (h)	No. (%) of oocytes			No. (%) of oocytes developed to		
		Injected	Survived^b^	Fertilized^c^	2-cell^d^	4-cell^d^	Morula/Blastocyst^de^
N	0	97	77 (79)	74 (96)	72 (97)	55 (74)	27 (36)^f^
N	1	71	59 (83)	58 (98)	50 (86)	11 (19)	4 (7)^g^
BD	0	88	62 (70)	60 (97)	60 (100)	55 (92)	42 (70)^h^
BDA	0	80	56 (70)	56 (100)	49 (88)	31 (55)	13 (23)^i^
BD	1	72	59 (82)	57 (97)	56 (98)	44 (77)	34 (60)^j^
BD	3	70	51 (73)	50 (98)	43 (86)	37 (74)	26 (52)^j^

^a^N: Oocytes were not treated.

BD: Oocytes were treated with MG132 before and during ICSI.

BDA: Oocytes were treated with MG132 before, during, and 1 h after ICSI.

^b^Percentages based on the no. of oocytes injected.

^c^Percentages based on the no. of oocytes survived.

^d^Percentages based on the no. of oocytes fertilized. Embryonic development was estimated at day 1 (2-cell), day3 (4-cell) and day5 (Morula/Blastocyst) after ICSI (day 0).

^e^Significant difference at *P* < 0.05 (f vs. g and h, g vs. h, i, and j, h vs. i, i vs. j).

The survival rates of untreated and MG132-treated oocytes after ICSI were 79–83% and 70–82%, and 96–98% and 97–100% of the surviving oocytes were fertilized, respectively. In the untreated group, the rate of development into morula and blastocyst was significantly lower for oocytes cultured for 1 h before ICSI (7%) than that for oocytes injected with sperm immediately after collection (36%). However, no significant difference was found between the in vitro development of oocytes treated with MG132 for 0, 1, and 3 h before and during ICSI (70%, 60%, and 52%, respectively). Conversely, such embryonic development was significantly decreased for oocytes further treated with MG132 for 1 h after ICSI (23%).

We investigated the in vivo development of oocytes treated with MG132 before and during ICSI (Table 3). The 1- and 2-cell embryos derived from the oocytes treated with MG132 for 0, 1, or 3 h before and during ICSI were transferred into pseudopregnant female rats. Embryos derived from untreated oocytes cultured for 0 or 1 h before ICSI were transferred into pseudopregnant females to serve as a control.

**Table 3:** Suppressive effect of MG132 on the spontaneous activation of oocytes and the in vivo development of oocytes injected with sperm.

MG132 treatment^a^	Culture time (h)	No. (%) of		
		Embryos transferred	Implantation sites^b^	Offspring^bc^
N	0	74	37 (50)	20 (27)^d^
N	1	68	15 (22)	7 (10)^e^
BD	0	67	30 (45)	21 (31)^d^
BD	1	64	27 (42)	20 (31)^d^
BD	3	71	10 (14)	6 (8)^e^

^a^N: Oocytes were not treated.

BD: Oocytes were treated with MG132 before and during ICSI.

^b^Percentages based on the no. of embryos transferred.

^c^Significant difference at *P* < 0.05 (d vs. e).

In the untreated groups, the developmental rate of embryos derived from oocytes cultured for 1 h (10%) was significantly lower than that of oocytes injected with sperm immediately after collection (27%). However, there was no significant difference in the developmental rate of embryos derived from oocytes treated with MG132 for 0 and 1 h before and during ICSI (both 31%). Offspring were obtained from embryos derived from oocytes treated with MG132 for 3 h before and during ICSI; however, the rate of live birth was low (8%).

### Chromosome alignment of oocytes after in vitro culture with MG132

**Figure 3:**
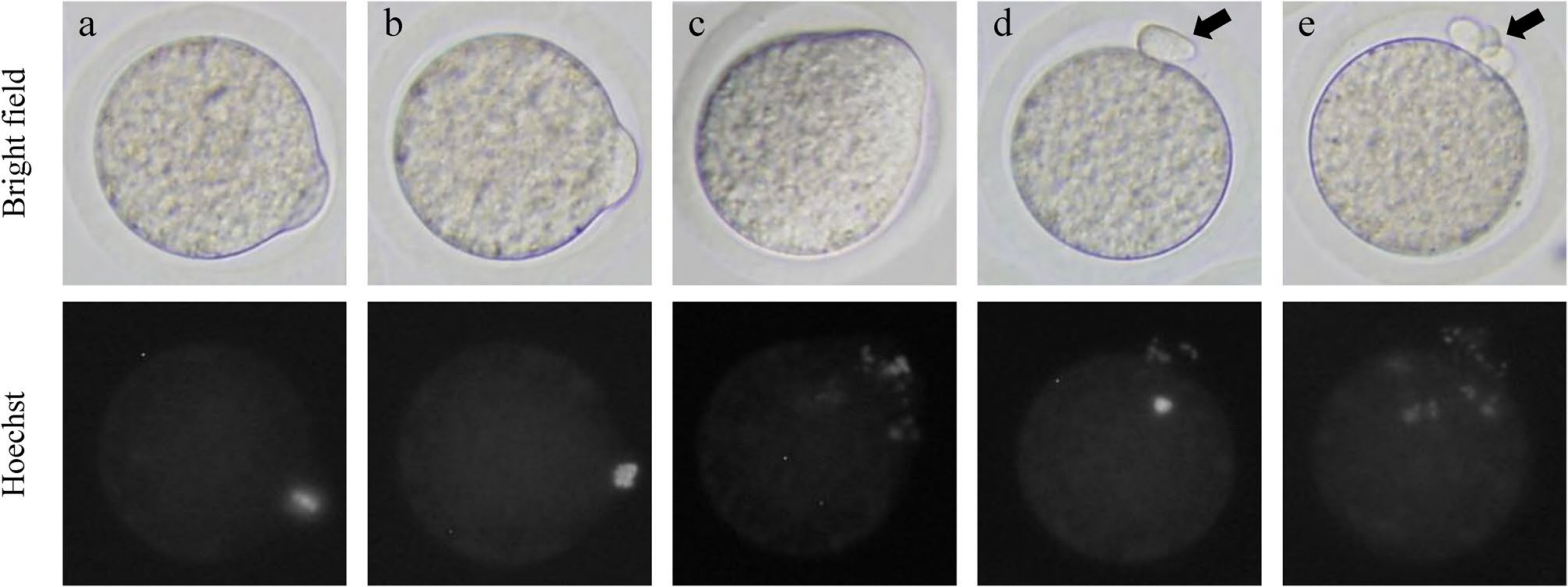
Chromosome alignment of oocytes after culture with or without MG132. The chromosomes in oocytes were stained with Hoechst 33,432. (**a**) MG132-treated oocyte with correctly aligned chromosomes. (**b**) MG132-treated oocyte with unaligned chromosomes. (**c**) MG132-treated oocyte with scattered chromosomes at 3 h of culture. d) Untreated oocytes at 1 h of culture. Chromosomes were completely separated. (**e**) Untreated oocytes at 3 h of culture. One set of separated chromosomes were scattered throughout the cytoplasm. Arrows point to the second polar body.

**Figure 4:**
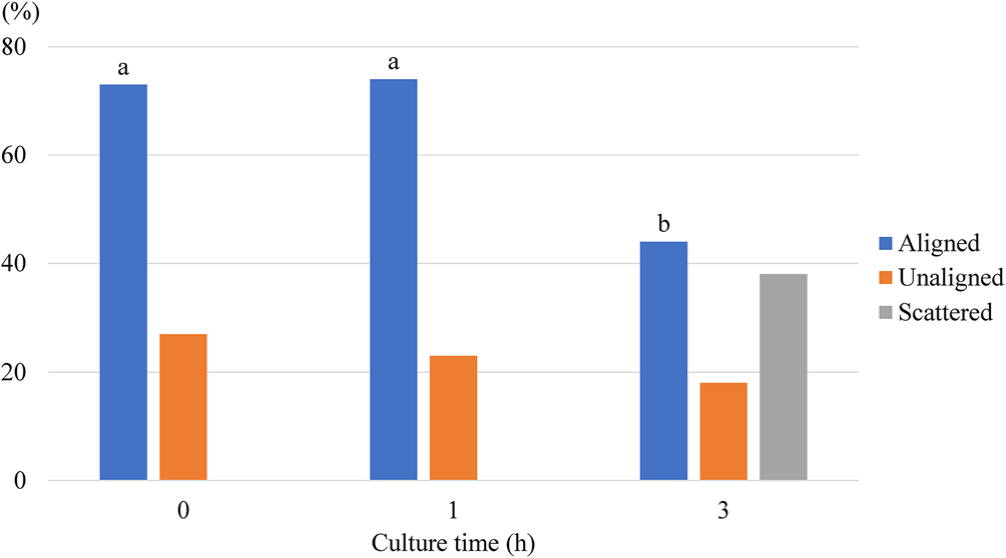
Effect of in vitro culture time on the chromosome alignment of oocytes treated with MG132. Aligned: Oocytes with correctly aligned chromosomes. Unaligned: Oocytes with unaligned chromosomes. Scattered: Oocytes with scattered chromosomes. Significant difference at *P* < 0.05 (a vs. b).

We examined the chromosomal alignment of oocytes cultured in vitro with MG132. Oocytes at 0, 1, or 3 h after culture with MG132 were stained with Hoechst 33,342. The chromosomes in these oocytes were then observed (n = 30–34). Oocytes were divided into three categories (Fig. 4): oocytes with correctly aligned chromosomes (Fig. 3a), unaligned chromosomes (Fig. 3b), and scattered chromosomes (Fig. 3c). The chromosomes in oocytes at 1 or 3 h after culture without MG132 were also stained and observed.

When oocytes were treated with MG132, there was no significant difference between the rates of oocytes with correctly aligned chromosomes at 0 and 1 h of culture (73% and 74%, respectively). However, this rate was significantly decreased in oocytes cultured for 3 h (44%). Further, oocytes with scattered chromosomes were observed at this time point (38%). In the untreated oocytes, chromosomes were completely separated at 1 h of culture (Fig. 3d), and one set of separated chromosomes was scattered throughout the cytoplasm at 3 h of culture (Fig. 3e).

## Discussion

We demonstrated that MG132 suppresses the spontaneous activation of rat oocytes before and during ICSI and increases the subsequent embryonic development. Once rat oocytes are collected from the oviduct, spontaneous activation immediately occurs^17–20^, which is harmful to the embryonic development after ICSI^22^. The novel ICSI protocol using MG132 is effective for obtaining stable rat offspring.

First, we determined the effect of the time from the hCG injection to ICSI on the embryonic development from oocytes. In superovulated mature rats, ovulation was reported to begin at 10–12 h after hCG injection, with almost full completion at 18 h after hCG injection^28^. If oocytes residing in the oviduct were not fertilized during the optimal period after ovulation, oocyte aging occurs in the ovulated oocytes, and aged oocytes display a low fertilization rate and poor developmental ability^29^. In this study, the developmental ability of oocytes was maintained until 22 h after hCG injection, but significantly decreased at 26 h after hCG injection (Table 1).

After retrieval from the oviducts, 98% of oocytes spontaneously activated and excluded the second polar body in culture within 1 h (Fig. 1). No oocytes formed pronuclear and fragmented oocytes were observed in this study. Furthermore, the in vitro and in vivo development of oocytes cultured for 1 h before ICSI was significantly decreased compared to that of the control (Tables 2 and 3). Spontaneous activation has been observed in various rat strains, despite the occurrence of strain-dependent differences in the incidence of activation^30^. The decrease in MPF activity of oocytes after retrieval from the oviduct triggers spontaneous activation^25^. Spontaneously activated oocytes extrude the second polar body without pronuclear formation, and the chromosomes are scattered in their cytoplasm^17,31^. In this study, scattered chromosomes were observed in spontaneously activated oocytes (Fig. 3e). However, most of oocytes in all culture condition survived, fertilized and developed to 2-cell stage (Table 2). This reason that the early-stage embryo is almost dependent on the accumulated maternal mRNA in the oocyte before MG132 treatment. After fertilization, sperm provide DNA and activate oocytes. However, sperm components play no major role in early stage embryogenesis^32^. Subsequent changes in oocytes after spontaneous activation, including chromosomal abnormalities, may adversely affect the late embryonic development from oocytes injected with sperm.

MG132 suppressed the spontaneous activation of oocytes before and during ICSI. And the embryonic development of these embryos was high (Tables 2 and 3). MG132 was inhibit proteasome activity by binding active site of proteasome. Inhibit of proteasome activity suppressed degradation of cyclin B. Spontaneous activation suppressed to maintain a high level of MPF activity in oocytes by existence of cyclin B^23,24^. In a previous report, the embryonic development from parthenogenetic rat oocytes cultured with MG132 for 1 to 4 h was high^33^. MG132 also improved the efficiency of somatic cell nuclear transfer in rats by suppressing the meiotic resumption of recipient oocytes^24–26^. MG132 could increase the embryonic development from rat oocytes injected with sperm by suppressing the spontaneous activation of oocytes after retrieval from the oviduct.

When oocytes were treated with MG132 before, during, and 1 h after ICSI, the in vitro development of oocytes injected with sperm was significantly decreased (Table 2). Although, MG132 are used as proteasome inhibitors to maintain rat oocytes at MII, its affect the integrity of meiotic spindle and influence subsequent oocyte development^33^. It is also required oocytes resume to activate immediately after ICSI for the normal development.

Of 73% and 74% of oocytes cultured with MG132 for 0 and 1 h, chromosomes were correctly aligned at the metaphase plate (Figs. 3a, 4). However, oocytes with scattered chromosomes significantly increased at cultured for 3 h (Figs. 3c, 4). Such finding suggests that correct chromosome alignment is necessary for the normal development of oocytes after ICSI as the developmental rate of embryos derived from oocytes treated with MG132 for 3 h before and during ICSI was decreased (Table 3). Although we employed an MG132 concentration of 5 μM based on previous reports^23,24^, another study reported that the meiotic spindles of rat oocytes treated with 10 μM MG132 were more stable than those treated with 5 μM MG132^34^. Further study of the optimal MG132 concentration in the media used for ICSI is required to improve the developmental ability of oocytes after ICSI by metaphase chromosome alignment.

In this study, MG132 suppressed the spontaneous activation of rat oocytes after retrieval from the oviducts and increased the embryonic development from oocytes injected with sperm. The novel ICSI protocol using MG132 is thus an effective method for obtaining stable rat offspring from sperm preserved as genetic resources.

## Methods

### Animals

Crlj:Wistar rats (Charles River Laboratories Japan, Inc., Kanagawa, Japan) were used in this study. All animals were allowed free access to food and water and were housed in plastic cages with controlled temperature (23 ± 3 °C), humidity (50 ± 10%), and lighting (7:00–19:00). All animal care procedures used in this study were carried out in accordance with ARRIVE guidelines under conformation with the Guidelines for Animal Experiments of Iwate University. The animal study was approved by the Animal Research Committee of Iwate University.

### Sperm freezing

Sperm were collected from male rats older than 14 weeks. Frozen sperm were used for ICSI instead of fresh sperm as rat sperm can be preserved efficiently using a simple solution without cryoprotection^12^. Briefly, two cauda epididymides of a male, which were separated from blood and fat, were placed in a plastic petri dish filled with 2.5 ml of a solution containing 10 mM Tris and 1 mM EDTA, adjusted to pH 8.0, (TE buffer; Thermo Fisher Scientific Inc., MA, USA). The cauda epididymides were minced using sharp scissors and left at room temperature for 5 min. The sperm was spread by shaking the dish, and 20-μl aliquots of the sperm suspension were dispensed into 1.2 ml cryotubes. The tubes were plunged directly into liquid nitrogen and stored until the ICSI.

### Oocyte collection

Female rats aged 9–16 weeks were used for oocyte collection. Females were superovulated via intraperitoneal injections of 300 IU/kg pregnant mare serum gonadotropin (PMSG, ASKA Animal Health Co., Ltd., Tokyo, Japan), and 300 IU/kg hCG (ASKA Animal Health Co., Ltd.) at 48 h after PMSG injection. Cumulus-oocyte complexes (COCs) were collected at 18, 22, or 26 h after hCG injection and incubated in mKRB containing 0.1% (w/v) hyaluronidase (Sigma-Aldrich, MO, USA) at 37 °C under 5% CO_2_ in air for 10 min. Oocytes freed from cumulus cells were washed three times with fresh mKRB.

When oocytes were treated with MG132 to suppress spontaneous activation, each medium for oocyte collection contained 5 μM MG132 (474,791, Sigma-Aldrich). The concentration of MG132 was determined to be 5 μM, based on previous research^23,24^.

### Evaluation of the second polar body extruded in the oocytes

Oocytes were collected at 18 h after hCG injection. The oocytes at the MII stage were cultured in 100 μl of mKRB with or without 5 μM MG132 at 37 °C under 5% CO_2_ in air. These oocytes were observed for 3 h of culture. Oocytes with the second polar body were recorded.

### ICSI

ICSI and the subsequent culture were performed as described previously by Kaneko et al.^35^ Frozen sperm were thawed at 37 °C for 1 min and 10-μl aliquots of the sperm suspension were diluted with 200 μl TE buffer. The diluted sperm suspension was sonicated using an ultrasonic homogenizer (VP-050, TAITEC Co., Saitama, Japan) to separate the sperm into head and tail.

Oocytes were placed in 5 μl media, and 1–2 μl sperm suspension was mixed with another 5 μl media containing 12% (w/v) polyvinylpyrrolidone. The zona pellucida of oocytes was drilled with a glass needle 3–3.5 μm in diameter using piezo pulses (speed: 2–4, intensity 1–2). A sperm head was hung on the tip of the needle and then injected into the oocyte (speed: 1, intensity: 1). ICSI was completed within 1 h.

Oocytes collected with MG132 were injected with sperm in the presence of 5 μM MG132. The injected oocytes were washed three times and kept in mKRB without MG132 at 37.5 °C under 5% CO_2_ in air for 10 min to remove MG132 from oocytes.

### Embryo culture and transfer

Oocytes that survived after ICSI were cultured in 100 μl of mKRB at 37.5 °C under 5% CO_2_ in air. Oocytes that formed two pronuclei were considered fertilized at 6 h after culture. The 1- and 2-cell embryos at 24 h after ICSI were transferred to 100 μl of modified Rat 1-Cell Embryo Culture Medium (mR1ECM)^36^, and cultured to blastocyst at 37.5 °C under 5% CO_2_ in air.

Proestrus females were mated with vasectomized males the day before embryo transfer. The 1-and 2-cell embryos at 24 h after ICSI were transferred into the oviducts of females with vaginal plugs^37^. The numbers of implantation sites and offspring were counted at 21 days of gestation.

### Time schedules of ICSI using MG132

ICSI using MG132 was performed according to the time schedule shown in Fig. 2. Oocytes were collected in mKRB with MG132 at 18 h after hCG injection. The oocytes were cultured in mKRB containing 5 μM MG132 at 37.5 °C under 5% CO_2_ in air. The oocytes cultured for 0, 1, or 3 h were injected with freeze-thawed sperm in the presence of 5 μM MG132. These oocytes were transferred into fresh mKRB without MG132 immediately after ICSI. Some oocytes after ICSI with MG132 were further cultured in mKRB containing 5 μM MG132 at 37.5 °C under 5% CO_2_ in air for 1 h, and transferred to mKRB without MG132. Oocytes collected in mKRB without MG132 were also injected with sperm after culture in mKRB at 37.5 °C under 5% CO_2_ in air for 0 or 1 h to serve as a control. These oocytes were cultured in vitro until the blastocyst stage.

Embryos derived from oocytes treated with 5 μM MG132 for 0, 1, or 3 h before and during ICSI were transferred into pseudopregnant female rats. Embryos derived from untreated oocytes injected with sperm after culture for 0 or 1 h were transferred into pseudopregnant females to serve as a control.

### Chromosome staining of oocytes

Oocyte collection, culture, and MG132 treatment were performed according to the same protocol used for extrusion of the second polar body. After culturing with MG132, oocytes with the second polar body were discarded, although only few oocytes extruded the second polar body. The oocytes cultured for 0, 1, or 3 h were stained with mKRB containing 10 μg/ml Hoechst 33,342 (FUJIFILM Wako Pure Chemical Corporation, Osaka, Japan) at room temperature in the dark for 10 min. The oocytes were washed three times and placed in fresh mKRB. The chromosomes in oocytes were observed under a fluorescence microscope. In addition, chromosomes in oocytes cultured without MG132 for 1 and 3 h were stained and observed.

## Data analysis

Each experiment was repeated at least three times. All data were compared using the chi-square test, followed by Ryan’s multiple comparison test.

## Data Availability

The authors declare that the data supporting the findings of this study are available within the paper.
